# Transcription Factors Targeted by miRNAs Regulating Smooth Muscle Cell Growth and Intimal Thickening after Vascular Injury

**DOI:** 10.3390/ijms20215445

**Published:** 2019-10-31

**Authors:** Levon M. Khachigian

**Affiliations:** Vascular Biology and Translational Research, School of Medical Sciences, Faculty of Medicine, University of New South Wales, Sydney NSW 2052, Australia; L.Khachigian@unsw.edu.au; Tel.: +61-2-9385-2537

**Keywords:** miRNA, transcription factors, immediate-early genes, smooth muscle cells, intimal thickening, neointima formation

## Abstract

Neointima formation after percutaneous coronary intervention (PCI) is a manifestation of “phenotype switching” by vascular smooth muscle cells (SMC), a process that involves de-differentiation from a contractile quiescent phenotype to one that is richly synthetic. In response to injury, SMCs migrate, proliferate, down-regulate SMC-specific differentiation genes, and later, can revert to the contractile phenotype. The vascular response to injury is regulated by microRNAs (or miRNAs), small non-coding RNAs that control gene expression. Interactions between miRNAs and transcription factors impact gene regulatory networks. This article briefly reviews the roles of a range of miRNAs in molecular and cellular processes that control intimal thickening, focusing mainly on transcription factors, some of which are encoded by immediate-early genes. Examples include Egr-1, junB, KLF4, KLF5, Elk-1, Ets-1, HMGB1, Smad1, Smad3, FoxO4, SRF, Rb, Sp1 and c-Myb. Such mechanistic information could inform the development of strategies that block SMC growth, neointima formation, and potentially overcome limitations of lasting efficacy following PCI.

## 1. Introduction

Vascular SMCs are non-terminally differentiated, in the sense that these cells have the capacity to undergo a change in phenotypic state. A shift from their normally “contractile” to “synthetic” phenotype in response to altered local environmental cues, such as injury, can lead to proliferation, migration and reduced expression of SMC-specific differentiation marker genes [1,2]. miRNAs can regulate this plasticity, also known as “phenotype switching” [3,4]. This switch is a feature of PCI (also known as angioplasty or stenting), which can lead to restenosis or in-stent restenosis months after the procedure [5]. Phenotypic switching can also underpin the pathogenesis of atherosclerosis, aortic aneurysm formation and hypertension [6,7,8]. While drug-eluting stents, and more recently, biodegradable drug-eluting stents, have improved clinical outcomes following PCI [9,10], challenges such as in-stent SMC hyperplasia, inadequate re-endothelialization and thrombosis remain [11,12]. Hence, a better understanding of molecular control in injured blood vessels and effective translation of this knowledge into the clinical arena is needed.

## 2. miRNA Biogenesis

Work conducted over the last decade has established that the molecular and cellular response to vascular injury or stress is, at least in part, controlled by miRNAs [13]. miRNAs are a class of conserved, small (~22 nucleotides) non-coding RNAs located throughout the genome in intergenic or intronic sequences [14,15]. miRNAs represent one of the most abundant gene families in animals, plants and viruses [16]. miRNAs are key regulators of post-transcriptional gene regulation. A single miRNA can potentially impact hundreds or even thousands of genes simultaneously [17]. Over 60% of human protein-coding genes have at least one conserved miRNA-binding site [18]. miRNA biogenesis involves the processing of the DROSHA ribonuclease III in the nucleus, the endoribonuclease DICER in the cytoplasm, then loading onto Argonaute (binding modules that accommodate small RNA in the form of RISC) and RNA decay [19]. However, it starts with polymerase II-dependent transcription from miRNA promoters. Some miRNAs have their own promoters, while others are embedded in introns of protein-coding genes. miRNAs bind to target mRNA for degradation and the inhibition of translation [20,21], and form the target recognition component of RISC [22]. It is generally understood that miRNAs guide RISC to target mRNAs by way of the seed pairing rule [23]. miRNAs have pleiotropic effects. For example, some miRNAs can serve as tumor suppressors, whereas others can promote tumor growth and metastasis [24]. A range of environmental factors (such as alcohol, drugs, smoking, pathogens, radiation and stresses) can regulate miRNA expression, and have been the subject of computational modelling [25,26]. miRNAs can regulate vascular pathobiology by direct and indirect effects on gene expression, as reviewed elsewhere [27].

## 3. miRNA Control of Transcription Factors Regulating SMC Proliferation and Intimal Thickening

This article reviews the role of miRNA in molecular and cellular processes regulating neointima formation, typically involving SMC accumulation, with particular focus on transcription factors (Table 1). Certain transcription factors are the products of IEGs, transiently and rapidly activated in response to injury, and govern rates and patterns of gene expression and phenotype in SMCs. For example, the IEG-encoded transcription factors Egr-1, junB and KLF4 are promptly induced by injury, and a range of other pathophysiologically relevant stimuli [28,29,30,31]. Among a range of rodent models used to study SMC growth and neointima formation [32] is that involving balloon catheter injury of rat carotid arteries, as developed by Clowes and Reidy in the early 1980s [33,34,35,36]. 

### 3.1. miR-145 and miR-143

miR-145 and miR-143 are co-transcribed from a single bicistronic unit [37], and control intimal thickening after vascular injury. miR-145 [38] and miR-143 [39] overexpression by adenovirus inhibits SMC proliferation and neointima formation in rat carotid arteries 14 days after balloon injury. miR-143 and miR-145 target a network of mediators that facilitate SMC differentiation and inhibit proliferation. Elia and colleagues knocked out miR-143(145) in mice and noted that SMCs have a dedifferentiated, migratory, and proliferative state [40]. miR-143 and miR-145 control KLF4, KLF5, Elk-1, Sp1 and myocardin [5,37,38]. KLF4 is a zinc finger transcription factor transiently expressed following vascular injury [41]. KLF4 levels increase in rat carotid arteries [31] with kinetics similar to those of Egr-1 and AP-1 family members, c-jun, c-fos, junB, junD and fosB, notably within minutes after injury [28,42,43]. At least two groups of investigators have recognized the capacity of circulating miR-143 to serve as a clinical biomarker for predicting in-stent restenosis in patients with lower extremity arterial occlusive disease [44] or coronary artery disease [45]. Others have found that miR-143/-145 levels are reduced in patients with essential hypertension when compared with healthy subjects [46,47]. miR-145, at least, has therapeutic potential. Muthiah and colleagues coated stents with miR-145 and a biocompatible polysorbitol-based osmotically active transporter was used to facilitate intracellular delivery suppressing SMC proliferation and reduced expression of target proteins [48]. 

### 3.2. miR-146a and miR-92a

miR-146a, on the other hand, promotes SMC proliferation by targeting KLF4 [49]. Sun et al. found that miR-146a overexpression reduced KLF4 levels and stimulated SMC growth, whereas miR-146a inhibition increased KLF4 levels, and inhibited SMC proliferation and intimal thickening in injured rat carotid arteries. The investigators found that transcription of miR-146a is regulated by KLF4 and KLF5, and that miR-146a and KLF4 control the expression of the other. KLF4 and KLF5 are recruited to the miR-146a promoter where they compete with one another to control promoter activity [49]. Iaconetti and colleagues found that KLF4 and MKK4 are targeted by miR-92a in EC and that antagomiR-92a can stimulate endothelial migration, proliferation and re-endothelialization in injured arteries, and inhibit intimal thickening with or without stenting. miR-92a inhibition also enhanced endothelial NOS expression [50], while NO production limited intimal hyperplasia as reported by Indolfi et al. [51]. 

### 3.3. miR-200c

Zheng and colleagues conducted a miRNA array screen involving 200 unique mature human miRNAs and found that miR-200c was reduced in vascular hyperplasia [52]. miR-200c targets KLF4 and Ubc9, a SUMO-conjugating enzyme. Ubc9 stimulates the SUMOylation of KLF4, which facilitates the recruitment of transcriptional corepressors (such as HDAC2 and NCoR) to the p21 and miR-200c promoters. Reduced levels of miR-200c allowed Ubc9 and KLF4 expression, thus suppressing miR-200c and increasing SMC growth. These investigators proposed the existence of a miR-200c-SUMOylated KLF4 feedback loop during SMC proliferation [52]. While miR-200c and KLF4 appear to cross-regulate, the study stopped short of delineating how KLF4-miR-200c controls SMC proliferation.

### 3.4. miR-663

SMC phenotype is also controlled by miR-663. Li and colleagues found that miR-663 negatively regulates SMC migration and differentiation marker gene expression, and that this involves the inhibition of junB and downstream genes including, MYL9 and MMP-9. Licht et al. had earlier shown that junB controls cell motility and vascular contractility in mice through its regulation of MYL9 transcription [53]. Li’s group found that growth factor-induced SMC proliferation and migration is reduced by siRNA knockdown of junB that increased expression of SMC contractile genes. miR-663 overexpression reduced junB expression, increased SMC differentiation marker gene expression and inhibited intimal thickening in mice following carotid artery ligation. Modified miR-663 expression did not seem to impact the expression of KLF4, fosB, myocardin and CEBPB [54].

### 3.5. miR-140-3p

Zhu and colleagues demonstrated a functional role for miR-140-3p in SMC hyperplasia. miR-140-3p inhibits SMC proliferation, stimulates apoptosis and reduces intimal thickening by targeting c-Myb and Bcl-2 [55]. With antisense oligonucleotides, Simons et al. had decades earlier, demonstrated that c-Myb regulates intimal SMC accumulation [56]. c-Myb regulates Bcl-2 [57], and both are targets of miR-140-3p [55]. Zhu and colleagues discovered miR-140-3p levels are reduced in arterial tissue from patients with peripheral artery disease and in-stent restenosis [55]. Lentiviral delivery of miR-140-3p caused an approximate 50% reduction in intimal hyperplasia after injury, and inhibited c-Myb and Bcl-2 without impeding re-endothelialization [55]. The unknown impact of miR-140-3p on other target genes and other cell types was a noted potential limitation in this study.

### 3.6. miR-191

We evaluated the effects of a mimic precursor of miR-191 on neointima formation in rat carotid arteries following injury. miR-191 inhibited neointima formation compared with precursor mimic miRNA control after injury. miR-191 suppressed Egr-1 expression and reduced Ki-67 staining [58]. Egr-1 positively regulates intimal thickening after balloon injury [42,59,60] and permanent ligation [61] in rat arteries, vein-to-artery grafts in rabbits [62] and porcine arteries following coronary stenting [63]. Egr-1 regulation is not confined to acute vascular injury. For example, Egr-1 controls pulmonary vascular remodeling and neointimal lesions in flow-associated pulmonary arterial hypertension [64]. Egr-1 and dependent genes are expressed in human and mouse atherosclerosis [65]. Albrecht et al. found that Egr-1 deficiency in bone marrow-derived cells reduced the formation of atherosclerotic lesions [66]. Interestingly, miR-191 levels are reduced in sera or plasma of patients with AMI [67,68] and T2DM [69]. 

### 3.7. miR-22-3p

There is considerable literature linking miRNAs with transcription factors not classified as IEGs in intimal hyperplasia. For example, Huang and colleagues found that miR-22-3p regulates SMC proliferation and migration by targeting HMGB1 [70], a highly conserved nuclear protein that enhances transcription and also serves as a pro-inflammatory cytokine after it is released into the extracellular milieu [71,72]. The miR-22-3p levels are lower in arteries from arteriosclerosis obliterans (an occlusive vascular disease mainly affecting the abdominal aorta and lower extremities), when compared with normal arteries, and negatively correlate with HMGB1. Lentiviral delivery of miR-22-3p suppresses HMGB1 and intimal hyperplasia in balloon-injured rat carotid arteries [70] implicating HMGB1 as a therapeutic target. 

### 3.8. miR-26a and miR-23b

The Smad family of signal transducers and transcriptional modulators regulate SMC phenotype [73]. Yang and colleagues found that miR-26a targets Smad1, and in doing so, serves as a positive regulator of PDGF-BB-mediated SMC phenotypic transition. Smad1 expression was reduced in injured rat carotid arteries following PDGF-BB exposure. In contrast, miR-26a levels increased in SMCs exposed to PDGF-BB, and in rat arteries following injury. SMC differentiation marker gene (e.g., SM-MHC, calponin) expression that decreased by treatment with PDGF-BB, was partly abrogated by transfection of anti-miR-26a. miR-26a provides a regulatory link between PDGF and the BMP/TGF-β family [74]. Iaconetti et al. found that miR-23b targets a different Smad and regulates the SMC phenotype. miR-23b overexpression inhibits SMC growth and migration, and promotes ACTA2 and SM-MHC expression. Adenoviral miR-23b overexpression reduces intimal hyperplasia, in part, by suppressing Smad3 and transcription factor FoxO4 [75]. Recent studies have shown that miR-23b is negatively regulated by long noncoding RNA XR007793, which is inducibly expressed in injured carotid arteries. XR007793 knockdown can repress SMC migration and proliferation [76]. 

### 3.9. miR-125a-5p

Smads, like many other transcription factors, cooperate with members of the Ets family (e.g., [77]). Ets-1 is a direct target of miR-125a-5p in SMCs. Gareri and colleagues found that miR-125a-5p expression decreases in carotid arteries within 3–7 days of injury and is reduced in SMCs exposed to PDGF-BB. Vascular injury and PDGF-BB both induce Ets-1 expression. miR-125a-5p overexpression reduces SMC proliferation and migration, and stimulates the expression of SMC markers ACTA2, SM-MHC and SM-22α. miR-125a-5p downregulation in response to PDGF-BB, coincided with the loss of SMC contractile phenotype [78]. Although this study provides important evidence that miR-125a-5p regulates vascular remodeling, whether other miRNAs work with miR-125a-5p to modulate the SMC phenotype remains unresolved.

### 3.10. miR-125b

Ubiquitously expressed SRF binds CArG elements with myocardin, a cofactor in the promoter regions of genes typifying SMC differentiation including ACTA2 and SM-MHC [5,79,80]. Recent studies using atomic force microscopy showed that the SRF/myocardin pathway provides an important mechanism regulating SMC stiffness, and mediates increased aortic stiffness associated with hypertension [81]. Chen and colleagues recently used a lentiviral transduction system to overexpress miR-125b (targeting SRF) to inhibit neointima formation in injured rat carotid arteries. miR-125b expression was lower in arteriosclerosis obliterans arteries and PDGF-BB stimulated SMCs. They found that miR-125b inhibits SMC migration and proliferation, and stimulates SMC apoptosis [82] suggesting control of SMC function through the miR-125b/SRF pathway.

### 3.11. miR-17

Recent studies by Yang et al. [83] determined that miR-17 promoter activity in SMCs is positively regulated by NF-κB p65 signaling, and also suppresses Rb protein expression. Interestingly, miR-17 promotes SMC growth and stimulates G_1_/S transition. miR-17 targets the 3’ untranslated region in Rb mRNA. p65 inactivation caused decreased expression of miR-17. SMC proliferation may be controlled by a distinct mechanism involving p65, miR-17 and Rb [83]. Another group had earlier detected higher levels of miR-17-5p in plasma from patients with CAD compared with healthy or those with non-significant CAD, and suggested miR-17-5p may serve as a potential biomarker of severity for coronary atherosclerosis [84].

**Table 1:** Transcription factor regulation by miRNA in response to vascular injury.

miRNA	Target	Reference
miR-17	Rb	[83]
miR-22	HMGB1	[70]
miR-23b	Smad3, FoxO4	[75]
miR-26a	Smad1	[74]
miR-92a	KLF4	[50]
miR-125a	Ets-1	[78]
mIR-125b	SRF	[82]
miR-133	Sp1	[39]
miR-140	c-Myb	[55]
miR-145, miR-143	KLF4, KLF5, Elk-1	[37,38]
miR-146a	KLF4	[49]
miR-191	Egr-1	[58]
miR-200c	KLF4	[52]
miR-663	junB	[54]

### 3.12. miR-133

miR-133 is yet another miRNA linked with the SMC phenotype. Torella and colleagues found that adenoviral overexpression of miR-133 reduces intimal thickening after injury, whereas anti-miR-133 enhances SMC growth and neointima formation. miR-133, which is regulated by ERK, inhibits SMC proliferation by repressing Sp1 [39]. Sp1 is a zinc finger transcription factor that typically controls the basal expression of many genes including PDGF-A [85] and PDGF-B [86]. miR-133 reduced, while anti-miR-133 increased Sp1 levels in SMCs [39]. miR-133 also decreased levels of CNN1, TAGLN2 and ACTA2, yet increased SM-MHC [39]. Deaton et al. previously showed that Sp1 regulates the KLF4 promoter in SMCs exposed to PDGF-BB [87]. 

In a recent study of 111 patients, De Rosa and colleagues discovered an association between the incidence of adverse cardiac events and the transcoronary concentration gradients of miR-133a measured by PCR. There was significant association with increased rates of death in patients with acute coronary syndromes and incidence of revascularization due to in-stent restenosis [88]. Future studies in larger cohorts with more sophisticated miRNA quantitation techniques should help further establish the importance of circulating miRNA, as biomarkers in vascular disease.

## 4. Therapeutic Implications

The findings above illustrate that transcription factor expression profiles in vascular SMCs are regulated by patterns of miRNA that change in response to injury. Whether specific miRNAs can serve as disease biomarkers in one or more cardiovascular indications will depend on findings in greater and more diverse patient populations. Strategies effecting endogenous miRNA control are being developed as potential therapeutics. Such approaches include the use of miRNA sponges (which can inhibit families of miRNA [89]), miRNA-masks (which block the access of target miRNA to binding sites, thereby perturbing miRNA function [90]), small molecule inhibitors (such as aryl amide derivatives that inhibit miR-21 [91]), antisense oligonucleotides (typically chemically modified for increased stability, such as miravirsen (SPC3649), which inhibits miR-122 biogenesis [92] and is used clinically for hepatitis C virus [93,94]), and miRNA mimics (such as synthetic miR-34 mimics [95]). While miRNA therapeutics have progressed in clinical trials for settings including infectious disease [96] and cancer [97], these have not yet been evaluated in PCI, and challenges remain. For example, Phase I trials of RGLS4326 (anti-miR-17) for inherited polycystic kidney disease, RG-101 (anti-miR-122) for hepatitis C virus, and MRX34 (miR-34 mimic) were stopped in light of unexpected toxicity or adverse events [98]. miRNA therapeutics currently in Phase I/II include MRG-110 (anti-miR-92a) for heart failure incisional complications, MRG-106/cobomarsen (anti-miR-155) for lymphoma/leukemia, and MRG-201/remlarsen (miR-29 mimic) for cutaneous fibrosis. Key to the ultimate success of efficacious miRNA therapeutics is efficient delivery, stability in vivo and minimal off-target effects.

## 5. Concluding Remarks

miRNAs can undergo altered expression following vascular injury and control the SMC phenotype and regulate differentiation or de-differentiation. While it is clear that interactions between miRNAs and transcription factors can shape regulatory networks, our understanding of the complexity and balance of this process is far from complete. Further mechanistic information in models of vascular disease will provide new insights into how such networks are controlled. This should underpin therapeutic strategies in PCI and help identify and exploit the diagnostic and prognostic value of miRNA.

## Abbreviations

ACTA2	SMα-actin
AMI	acute myocardial infarction
BMP/TGF-β	bone morphogenetic protein/transforming growth factor-β
CAD	coronary artery disease
CEBPB	CCAAT enhancer binding protein β
CNN1	calponin
DICER	dsRNA endoribonuclease
DROSHA	Drosha ribonuclease III
EC	endothelial cell
Egr-1	early growth response-1
ERK	extracellular signal-regulated kinase
FoxO4	Forkhead box O4
IEG	immediate-early gene
HDAC2	histone deacetylase 2
HMGB1	high-mobility group box 1 protein
KLF4	Kruppel-like factor 4 (GKLF/EZF)
KLF5	Kruppel-like factor 5 (BTEB2/IKLF)
miRNA	microRNA
MKK4	mitogen-activated protein kinase kinase 4
MMP-9	matrix metalloproteinase-9
MYL9	myosin light chain 9
NCoR	nuclear corepressor
NO	nitric oxide
NOS	nitric oxide synthase
PCI	percutaneous coronary intervention
PCR	polymerase chain reaction
PDGF	platelet-derived growth factor
Rb	retinoblastoma
RISC	RNA-induced silencing complex
RNA	ribonucleic acid
Smad1	SMAD family member 1
Smad3	SMAD family member 3
SMC	smooth muscle cell
SM-22α	smooth muscle-22α
SM-MHC	SM-myosin heavy chain (MYH11)
SRF	serum response factor
SUMO	small ubiquitin-like modifiers
T2DM	type 2 diabetes mellitus
TAGLN2	transgelin-2
